# Evaluation of the haematinic, antioxidant and anti-atherosclerotic potential of *Momordica charantia* in cholesterol-fed experimental rats

**DOI:** 10.1016/j.toxrep.2022.03.042

**Published:** 2022-03-31

**Authors:** Silvanus Olu Innih, Ikechi Gerald Eze, Kingsley Omage

**Affiliations:** a Department of Anatomy, School of Basic Medical Sciences, College of Medical Sciences, University of Benin, Benin City, Nigeria; b Department of Biochemistry, College of Basic Medical Sciences, Igbinedion University, Okada, Edo State, Nigeria; c Division of Endocrinology, Diabetology and Nephrology, Department of Internal Medicine, University Hospital Tübingen, Germany

**Keywords:** GPx, Glutathione Peroxidase, ROS, Reactive Oxygen Species, SOD, Superoxide Dismutase, MDA, Malondialdehyde, CAT, Catalase, HMG-CoA, Hydroxylmehtylglutharyl Coenzyme A, TCA, Trichloroacetic Acid, TBA, Thiobarbituric Acid, LD, Lethal Dose, LDMC, Low Dose *Momordica charantia*, HDMC, High Dose *Momordica charantia*, RBC, Red Blood Cell, MCH, Mean Corpuscular Haemoglobin, MCHC, Mean Corpuscular Haemoglobin Concentration, MCV, Mean Corpuscular Volume, PCV, Packed Cell Volume, HCT, Haematocrit, WBC, White Blood Cell, PLT, Platelets, MPV, Mean Platelet Volume, PTAH, Phophostungstic Acid Haematoxylin, *Momordica charantia*, Atherosclerosis, Oxidative stress, Differential blood count, Haematocrit, Atorvastatin

## Abstract

**Background:**

*Momordica charantia* is popularly used in folk medicine in the management of hyperlipidemia and atherosclerosis.

**Purpose:**

To evaluate the anti-atherosclerotic potential of *M. charantia* as well as its haematinic and antioxidant potential.

**Methods:**

Seventy-two experimental rats were randomly assigned into 9 groups (I-IX) of 8 rats each. Group I (control), was given 1 ml distilled water; II received 250 mg/kg. *M. charantia*; III received 500 mg/kg *M. charantia*; IV was administered 100 mg/kg of Atorvastatin only; V was administered 30 mg/kg of cholesterol dissolved in coconut oil; VI was administered with 250 mg/kg of *M. charantia* plus 30 mg/kg of cholesterol. VII was treated with 500 mg/kg of *M. charantia* plus 30 mg/kg of cholesterol solution; VIII was administered 30 mg/kg cholesterol solution plus Atorvastatin at a dose of 100 mg/kg; IX was administered 1 ml of coconut oil only. After 60 days of administration, blood and aorta samples were obtained from the rats. The samples were subjected to biochemical, haematological and histological analysis using standard methods.

**Results:**

Glutathione peroxidase (GPx), Malondialdehyde (MDA) and Catalase (CAT) activities were significantly higher in the treated groups as compared to the control groups. There were significant increases in the monocyte counts of the groups given low dose (250 mg/kg) of the extract (LDMC), high dose (500 mg/kg) of the extract (HDMC), as well as atorvastatin. The mean corpuscular volume (MCV) and mean corpuscular haemoglobin (MCH) of the test groups administered were significantly higher than that of the control group. However, only the group administered with cholesterol plus HDMC showed significantly lower mean corpuscular haemoglobin concentration (MCHC) than that of the control group. Histological sections of the aorta show degeneration of the internal elastic lamina in the group fed with the diet only as well as vascular ulceration and stenosis in the aorta and heavy perivascular infiltrates of inflammatory cells. These alterations were however not visible in the groups administered with the extracts, as well as atorvastatin

**Conclusion:**

Our findings show the possible anti-atherosclerotic potential of the extract, which could be compared to that of the standard drug (atorvastatin).

## Highlights

• *M. charantia* is a medicinal herb whose extract exhibits anti-atherosclerotic properties.
• The herb maintained the functional and structural integrity of the aorta.
• It protects against oxidative stress and mediates inflammation.
• Its effects are comparable to that of the standard drug, Atovastatin.

## Graphical Abstract

Note Chol – Cholesterol, LDMC – Low Dose *Momordical charantia*, HDMC – High Dose *Momordica charantia*, SOD – Superoxide Dismutase, GPX – Glutathione Peroxidase, MDA – Malondialdehyde, CAT – Catalase, WBC – White Blood Cell, PLT – Platelets, MPV – Mean Platelet Volume, RBC – Red Blood Cell, Hb – Haemoglobin, PCV – Pack Cell Volume, MCV – Mean Corpuscular Volume, MCHC – Mean Corpuscular Haemoglobin Concentration, MCH – Mean Corpuscular Haemoglobin.

**Figure:**
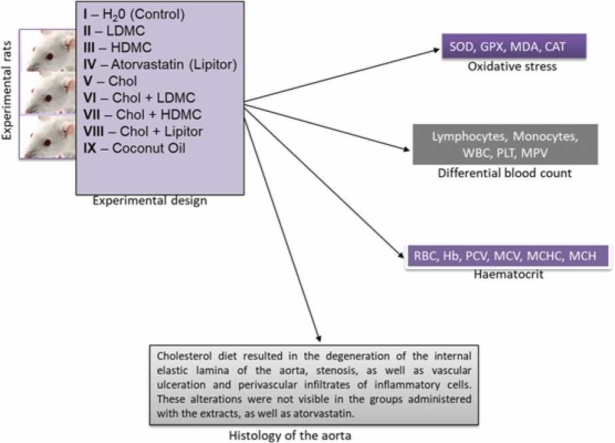

## 1 Introduction

Structural and functional changes of the walls of the blood vessels results in reduced vascular distension with increased arterial stiffness [6]. These changes associated with atherosclerosis underlie the pathogenesis of coronary, cerebral and peripheral vascular diseases which causes more morbidity and mortality in the Western World than any other disorder. Atherosclerosis often results from interplay of injuries to the walls of the blood vessels and inflammation [8]. Atherosclerotic plaques can be stable, where it produces symptoms related to chronic ischaemia by constricting the blood vessel lumens. It can also be unstable where it causes dramatic and potentially fatal ischaemic complications associated with acute plaque rupture, thrombosis or embolization [8].

Cardiovascular diseases associated with atherosclerosis could also result from oxidative stress or damage due to excessive production of free reactive oxygen species or radicals [21]. However, the oxidative damage could be prevented by the activities of antioxidants which are involved in the scavenging of the free radicals, thereby preventing them from reacting with biomolecules within the cells [29]. Antioxidants are known to mediate the treatment of atherosclerosis by either reducing the generation of free radicals, preventing the formation of atherosclerotic plaque and aggregation of platelets, prevention of mononuclear cell infiltration, or amelioration of endothelial dysfunction and vasodilation [21].

Treating or managing the changes and complications associated with atherosclerosis, with a view to reducing morbidity and mortality, is a major challenge in health care. Although synthetic drugs are potent, they often present undesirable side effects, high cost and low patient compliance. Medicinal plants are gaining much interest in recent times due to their use in ethnomedicinal intervention in treating or managing atherosclerosis and their therapeutic claims are now supported with sound scientific evidence [10]. *Momordica charantia* L. (Cucurbitaceae) popularly known as bitter melon, grows in different regions of Nigeria and in other tropical areas [18]. It is widely used in ethnomedicine for the management of hyperlipidemia and atherosclerosis [13], although a scientific support for this use is highly needed. Thus, this study was carried out to evaluate its effects in experimental rats fed with cholesterol diets with a view to providing scientific rationale for its use in ethnomedicine.

## 2 Materials and methods

### 2.1 Chemicals and reagents

Atorvastatin (Lipitor) (from Pfizer Pharmaceuticals, Great Britain), cholesterol powder, coconut oil, 50% ethanol, carbonate buffer, adrenaline solution, phosphate buffer, H_2_0_2,_ pyrogallol, TCA- TBA- HCl, phosphate buffer (pH 7.4), 6 M H_2_SO_4_, 0.01 M KMnO_4,_ EDTA, formalin, alcohol, xylene, paraffin wax, haematoxylin and eosin dyes, 1% acid alcohol, phosphotungstic acid haematoxylin (PTAH) stain. All the chemicals and reagents used, which are of analytical grades, were purchased from Sigma-Aldrich, Germany.

### 2.2 Collection of plant material and preparation of the plant extract

Freshly plucked leaves of *M. charantia* were obtained from Ovbiogie community in Ovia North-East Local Government Area of Edo State, Nigeria. The leaves were identified and authenticated in the Department of Plant Biology and Biotechnology (PBB), University of Benin, Benin City, Nigeria. The plant sample was assigned Herbarium Number UBHc0294 and a sample was deposited in the Herbarium of the department. The fresh leaves of the plant were air dried and pulverized into fine powder using an electric blender (pyeUnicam, Cambridge, Great Britain). The powdered sample was subjected to extraction using 50% ethanol. About 400 g of the powder was soaked in 50% ethanol for 72 h, with intermittent stirring. The mixture was afterwards filtered, using a muslin cloth, to obtain the extract. The dried ethanol extract was obtained by evaporation under reduced pressure using Rotary evaporator. It was then stored in an air-tight container and refrigerated at 4 °C for further use. The extract was freshly prepared every week till the end of the experiment.

### 2.3 Chemical characterization of *M. charantia*

High performance liquid chromatographic (HPLC) and chemical characterisations of *M. charantia* have reported the presence of flavonoids, phenols, alkaloids, saponins, tannin, glycosides and steroids [16], [17], [27].

### 2.4 Experimental animals, design and procedure

The experimental animals used for this study were adult male and female rats of the Wistar strain, weighing between 160 g and 180 g. They were bred in clean disinfected cages in the animal facility of the Department of Anatomy, University of Benin and allowed free access to food and water*.* Seventy- two of the experimental rats were randomly assigned into nine groups (I-IX) of eight rats each as follows; **Group I** (Control) rats were administered only 1 ml distilled water. The rats in **Group II** were administered 250 mg/kg ethanol extract of *M. charantia* leaf (LDMC) only. While **Group III** rats were administered 500 mg/kg ethanol extract of *M. charantia* leaf (HDMC) only. The rats in **Group IV** were administered 100 mg/kg of atorvastatin (lipitor) only. While the rats in **Group V** were administered freshly prepared cholesterol powder dissolved in 1 ml coconut oil (cholesterol solution) at a dose of 30 mg/kg body weight. The rats in **Group VI** were administered 250 mg/kg ethanol extract of *M. charantia* leaf (LDMC) and freshly prepared cholesterol solution at a dose of 30 mg/kg body weight. The rats in **Group VII** were administered 500 mg/kg ethanol extract of *M. charantia* leaf (HDMC) and freshly prepared cholesterol solution at a dose of 30 mg/kg body weight. **Group VIII** (Standard) rats were administered the standard drug atorvastatin (lipitor) at a dose of 100 mg/kg body weight and freshly prepared cholesterol solution at a dose of 30 mg/kg body weight. The rats in **Group IX** were administered 1 ml of coconut oil only. The various administrations were done orally and the treatment lasted for 60 days.

The coconut oil was used as the vehicle for cholesterol administration, while water (1 ml) was used as a vehicle for the administration of Lipitor and the extract. The cholesterol solution was administered for a period of 60 days to induce atherosclerosis in the experimental rats. Lipitor (from Pfizer Pharmaceuticals, Walton Oaks, Surrey, Great Britain) was used as a standard positive drug. After 60 days of administration, about 2 ml of blood samples were obtained by cardiac and aortic puncture from the heart and aorta of each rat into EDTA sample bottles. The aorta were also harvested from the experimental rats and dissected. The dissected organs were excised and promptly transferred into 10% formalin for fixation. The ethical approval for this research was obtained from the Research Ethics Committee of the College of Medical Sciences, University of Benin, Nigeria. The use of rats for the study was also according to the Ethical Guidelines Involving Whole Animal Testing of the Research Ethics Committee of the College of Medical Sciences, University of Benin, Nigeria.

### 2.5 In vivo assays

#### 2.5.1 Antioxidant assays

*Estimation of superoxide dismutase (SOD):* About of 0.2 ml of serum was mixed with 2.5 ml of carbonate buffer and 0.3 ml of freshly prepared adrenaline solution in a test tube (which served as test). Also, about 0.2 ml of distilled water was mixed with 2.5 ml of carbonate buffer and 0.3 ml of freshly prepared adrenaline solution in a test tube (which served as reference). The absorbance of the tests and reference were read at 420 nm. One unit of enzyme activity was taken as the amount of protein required to give 50% inhibition of adrenaline auto-oxidation. The results were expressed in units/ml [23].

*Estimation of glutathione peroxidase (GPx)*: To 0.2 ml of serum in a test tube, 2.5 ml of phosphate buffer, 2.5 ml of H_2_0_2_, 1.5 ml of pyrogallol were added. The reaction was allowed to stand for 30 mins at room temperature. A deep brown colour was formed, which was read at 430 nm [24]. The results were expressed in U/ml.

*Estimation of malondialdehyde (MDA):* To 0.1 ml of serum in a test tube, 2.0 ml of TCA- TBA- HCl (25% TCA, 1% TBA and 0.4 ml HCl) was added. The blank tube contained the same volume of reagents but 0.1 ml of distilled water in place of serum. The solution was heated in a water bath at 95 °C for 15 min. After cooling, the flocculent precipitate was removed by centrifuging at 1000 g for 10 min. The absorbance was read at 535 nm against the blank [5]. The results were expressed in μmol/l.

*Estimation of catalase (CAT):* To 0.5 ml of serum in a test tube, 5.0 ml of H_2_O_2_ was added. This was mixed by inversion and allowed to stand for 30 min. The reaction was stopped by addition of 1.2 ml of 50 mM phosphate buffer (pH 7.4), 1.5 ml of 6 M H_2_SO_4_ and 7 ml of 0.01 M KMnO_4_. The resulting solution was mixed by inversion and allowed to stand for 30 min. The absorbance was read at 480 nm within 30–60 s against distilled water. The absorbance of the enzyme blank was simultaneously measured with 1.0 ml of distilled water instead of H_2_O_2_ [7]. The results were expressed in μmol/min.

### 2.6 Hematological analysis

The blood samples were aspirated into the chamber of the Human Automated Hematology System Analyzer (ERMA PCE 210, ERMA, Japan) and diluted with isotonic saline solution. The parameters analyzed included hemoglobin (Hb), hematocrit (HCT), red blood cell (RBC), white cell count (WBC), and differential count (neutrophils, eosinophils, basophils, lymphocytes, and monocytes), platelets (PLT) and mean corpuscular volume (MCV).

Mean corpuscular hemoglobin (MCH) was calculated as:

$$\text{MCH}=\frac{\mathrm{Hb}(g/\mathrm{dL})x10}{\mathrm{RBC count}(\mathrm{million}/\mu L)}$$

Mean corpuscular hemoglobin concentration (MCHC) was calculated as:

$$\text{MCHC}=\frac{\mathrm{Hb x}10}{\mathrm{HCT}}$$

### 2.7 Histological procedure

The fixed tissues were completely dehydrated by passing them through ascending grades of alcohol. The tissues were left in 70% alcohol for 2 h, 90% alcohol for 18 h (overnight) and 100% alcohol for 4 h. The tissues were immersed in xylene for 5 h to allow complete removal of the alcohol. Infiltration of the tissues was carried out in an oven using molten paraffin wax at a temperature range of 56ºC to 60ºC with two changes at 15 min and 30 min. The molten paraffin wax was poured into an embedding mould and the infiltrated tissues placed in it in an orientation which allows both longitudinal and transverse cutting. After cooling of the molten paraffin wax, the tissue block was sectioned on a microtome (Leica RM 2235, UK) at 4 µm thickness. The sections were placed in 20% alcohol for spreading of the tissue. The ribbon-like sections were cut and floated in water bath at a temperature of 30ºC. The sectioned tissues were placed in xylene for 5 min for removal of paraffin wax. The tissues were passed through descending grades of alcohol (100%, 90% and 70%) and water for 5 min each for hydration. The tissues were stained in haematoxylin for 10 min and washed in water for 2 min. They were differentiated briefly in 1% acidified alcohol and washed in water. They were subsequently counterstained in eosin for 5 min and rinsed in 90% alcohol. Stained slides were viewed using an optical photomicroscope (Leica MC170 HD, Leica Bio systems, Wetzlar, Germany.) at × 100 and × 400 magnifications. Special stains used were elastic stain (ES) technique for elastic fibers and phosphotungstic acid haematoxylin (PTAH) stain for smooth muscle fibers [4].

### 2.8 Statistical analysis

Statistical analysis of the results was done by One Way Analysis of Variance (ANOVA) using IBM-SPSS statistics version 23 followed by Duncan's comparison test for significance (P < 0.05).

## 3 Results

**Fig. 1:**
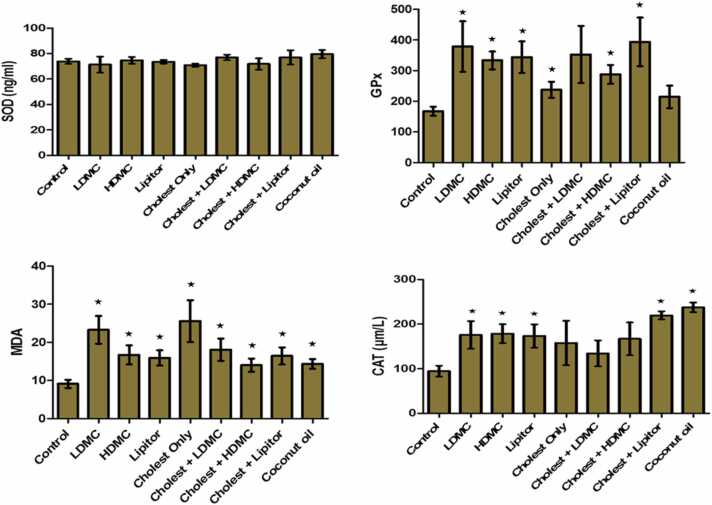
The effects of the different treatments on the activities of SOD, GPX, MDA and CAT (oxidative stress) of the experimental rats. N = 8. Cholest – Cholesterol, LDMC – Low Dose *Momordical charantia*, HDMC – High Dose *Momordica charantia*, SOD – Superoxide Dismutase, GPX – Glutathione Peroxidase, MDA – Malondialdehyde, CAT – Catalase.

In Fig. 1, the activities of SOD, GPx, MDA and CAT of the experimental animals were determined following the administration of cholesterol with *M. charantia* extract. We observed that there was no statistically significant difference (*p* > 0.05) across the groups for SOD. GPx activities were significantly (*p* < 0.05) higher in the treated groups as compared to the control groups and the groups administered with cholesterol and coconut oil only. The effect of the LDMC was similar to that of cholesterol + lipitor. All treated groups showed significantly (*p* < 0.05) higher MDA activities as compared with the control group. The activity was significantly (*p* < 0.05) lower in the HDMC, LDMC and cholesterol + lipitor groups as compared with the cholesterol only group. CAT activities were significantly (*p* < 0.05) increased in groups treated with LDMC, HDMC, atorvastatin, cholesterol with atorvastatin, and coconut oil only as compared with the control group. However, the CAT activity was significantly (*p* < 0.05) lower in the LDMC, HDMC and Lipitor groups compared to that in the cholesterol + lipitor and coconut oil group.

**Fig. 2:**
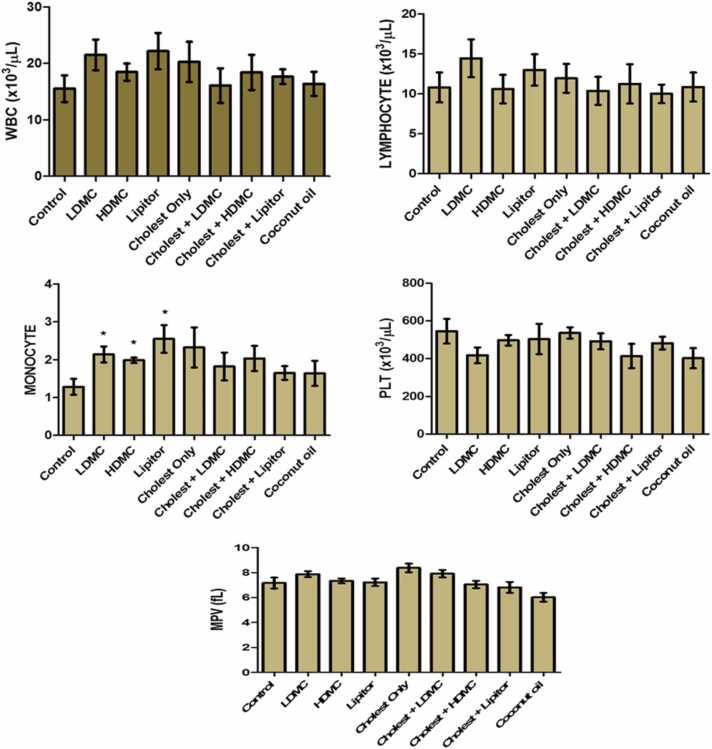
The effects of the different treatments on the differential blood count of the experimental rats. N = 8. Cholest – Cholesterol, LDMC – Low Dose *Momordical charantia*, HDMC – High Dose *Momordica charantia*, WBC – White Blood Cell, PLT – Platelets, MPV – Mean Platelet Volume.

In Fig. 2 , we determined the effects of the different treatments on the differential blood count of the experimental animals. It was observed that the treated groups showed no statistically significant differences in the white blood cell count, lymphocyte, platelet and mean platelet volume, as compared with the control group. However, there were significant (p < 0.05) increases in the monocyte counts of the groups given LDMC, HDMC, as well as atorvastatin, as compared to the control group.

**Fig. 3:**
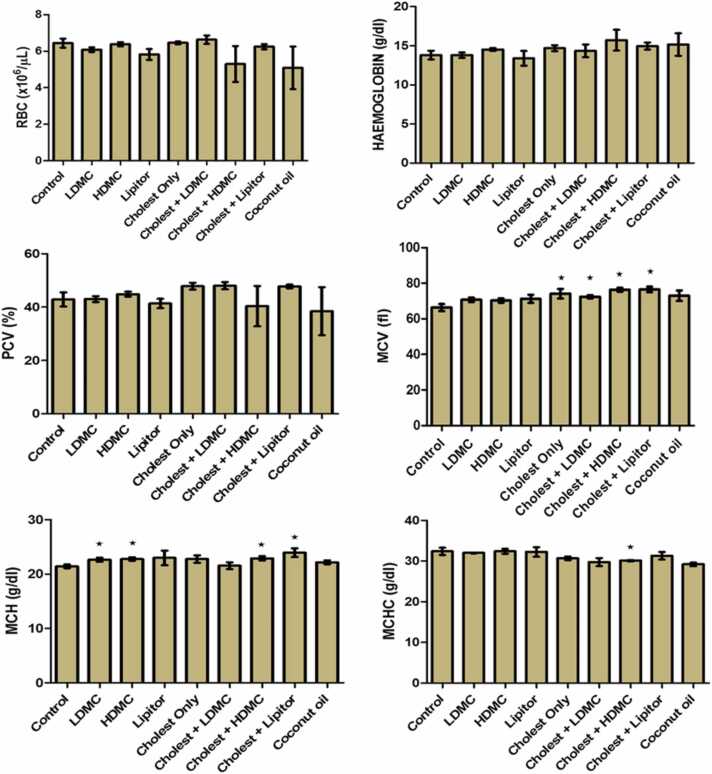
The effects of the various treatments on the haematocrit of the experimental rats. N = 8. Cholest – Cholesterol, LDMC – Low Dose *Momordical charantia*, HDMC – High Dose *Momordica charantia*, RBC – Red Blood Cell, Hb – Haemoglobin, PCV – Pack Cell Volume, MCV – Mean Corpuscular Volume, MCHC – Mean Corpuscular Haemoglobin Concentration, MCH – Mean Corpuscular Haemoglobin.

In Fig. 3, the effects of the various treatments on the haematocrit of the experimental animals were also examined. It was observed that the RBC, Hb and packed cell volume of the test groups did not differ significantly from those of the control group. The MCV of the experimental groups administered with cholesterol only, cholesterol plus LDMC, cholesterol plus HDMC and cholesterol plus atorvastatin showed significantly (*p* < 0.05) higher values as compared with that of the control group. Our results also show that the MCH was significantly (*p* < 0.05) higher in the groups administered with LDMC only, HDMC only, cholesterol plus HDMC and cholesterol plus atorvastatin, as compared with the MCH of the control group. However, only the group administered with cholesterol plus HDMC showed significantly (*p* < 0.05) lower MCHC than that of the control group.

**Fig. 4:**
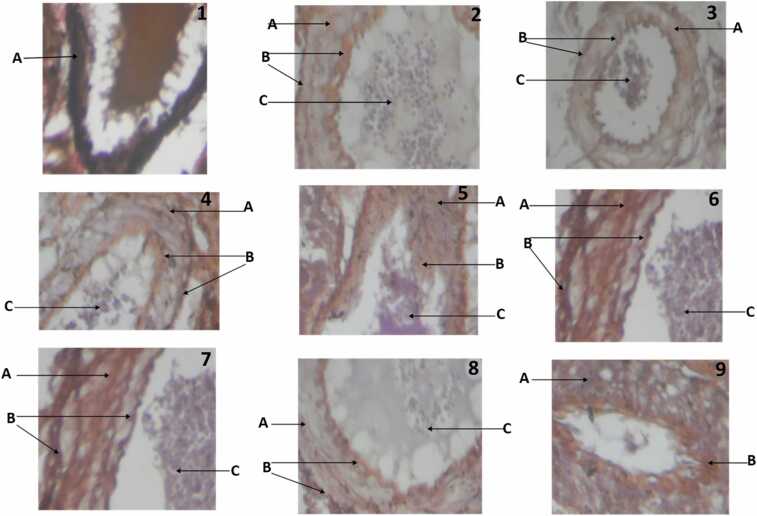
Aorta of the experimental rats stained with VVG (PTAH X 100). N = 8. RBC – Red Blood Cell. **1.** Control rat aorta showing normal elastic fibres + ++ (A). **2**. Rat given low dose extract only, showing collagen fibres + (A) and elastic fibres + + (B). **3**. Rat given high dose extract only, showing A, collagen fibres + (A), elastic fibres + + (B) and RBC + + (C). **4**. Rat given atorvastatin only showing, collagen fibres + (A), elastic fibres + + (B) and RBC + + (C). **5**. Rat given high cholesterol diet only showing, collagen fibres + (A), internal elastic lamina absent (B) and RBC + + (C). **6**. Rat given high cholesterol diet plus low dose extract showing, collagen + (A) elastic fibres + + (B) and RBC + + (C). **7**. Rat given high cholesterol diet plus high dose extract showing collagen + + (A), elastic fibres + + (B) and RBC + + (C). **8**. Rat given high cholesterol diet plus atorvastatin showing, collagen + + (A), elastic fibres + + (B) and RBC + (C). **9**. Rat given coconut oil only showing, collagen fibres + (A) and elastic fibres + + (B).

Fig. 4 shows the histological sections of the experimental rats after the different treatments. It was observed that cholesterol administration alone, at a dose of 30 mg/kg for a period of 60 days, resulted in the degeneration of the internal elastic lamina of the aorta, stenosis, as well as vascular ulceration and perivascular infiltrates of inflammatory cells. These alterations were however not visible in the groups administered with the extracts, as well as atorvastatin. In addition, administration of the extract as well as atorvastatin were shown to be effective at maintaining the integrity of the collagen and elastic fibres of the aorta.

## 4 Discussion

As a pre-experiment, we carried out acute toxicity study of the plant (*M. charantia*) by administering the ethanol extract orally to the experimental rats. Our result, although not included in the manuscript or published, revealed that the extract at a dose of 12,000 mg/kg caused neither obvious sign of toxicity nor mortality up to 14 days of observation. In addition, chronic administration of the extract did not significantly alter the total food intake of the treated animals. This is suggestive of the relative safety of the extract from the plant [25]. Studies on the HPLC analysis of the phytochemical constituents of *M. charantia* have reported the presence of flavonoids, phenols, alkaloids, saponins, tannin, glycosides and steroids in the plant [16], [17], [27].

It has been reported that certain phytochemicals, particularly flavonoids lower serum lipids by inhibiting the activity of HMG-CoA reductase and up-regulating the hepatic expression of alpha and gamma peroxisome proliferators. They prevent the oxidation of low-density lipoprotein, lower the blood levels of cholesterol, triglycerides, free fatty acids and phospholipids, thereby reducing the risk for the development of atherosclerosis [1], [3], [30]. It has long been suggested that consumption of tannin containing beverages especially green tea and red wines can cure or prevent a variety of disorders including heart related diseases [32]. Similarly, Han et al., [14] reported that saponins mediate anti-hyperlipidemic and hypocholesterolemic actions by inhibiting or delaying intestinal lipid absorption via a resin-like action and inhibiting pancreatic lipase activity. Also, it has been reported that saponins mediate their action via enhancing enterohepatic excretion of cholesterol in the bile acid [34].

Considering the importance of oxidative stress in the development and maintenance of atherosclerosis [20], we evaluated the effects of the various treatments on the oxidative parameters of the experimental rats. MDA, the indicator of lipid peroxidation was significantly elevated in cholesterol treated rats, suggesting the possible interactions of free radicals with unsaturated fatty acids in the membranes with resultant decrease in membrane permeability. This was however lowered significantly by the plant extract just as observed with atorvastatin. This finding is in agreement with previous report where *M. charantia* caused a reduction in the MDA level in diabetic experimental rats [1]. We observed that SOD level was not significantly affected in all the treated groups. This is an important antioxidant enzyme which scavenges superoxide anion. However, CAT which is responsible for detoxification of H_2_O_2_ produced by action of SOD and inhibits formation of superoxide radicals was found to be higher in the groups treated with both doses of the extract and atorvastatin. This suggests a similar activity between the extract and the standard drug (atorvastatin). GPx is also one of the main endogenous antioxidants and its depletion has been reported to result in increased mitochondrial ROS emission and loss of muscle force [9]. It is the substrate of the GPx reaction which scavenges H_2_O_2_ and GPx depletion sensitizes the pyruvate dehydrogenase complex or enzymes of the electron transport chain to elevate ROS production [14], [9]. Thus, decreased GPx, and consequently GPx levels, will contribute to heighten mitochondrial H_2_O_2_ emission, while increase in glutathione will result in decreased mitochondrial H_2_O_2_ emission. Our findings show that GPx activities were significantly higher in the experimental groups treated with the extract and the standard drug. This equally gives credibility to our suggestion of similar activity or effect between the extract and the standard drug.

Analysis of the differential blood count show an increase in the monocyte count in the groups treated with cholesterol and atorvastatin. The mean corpuscular volume or cell volume also increased in all the groups treated with cholesterol. The increased MCH in the groups treated with *M. charantia* and atorvastatin, shows a likely increase in oxygen carrying capacity, which is more significant following the administration of cholesterol plus high dose *M. charantia.* Our findings which shows a significant increase in leucocyte count is inconsistent with findings from studies on atherosclerosis of coronary arteries [15]. Moreover, we observed that monocytes were more abundant in groups treated with *M. charantia* only which is also contrary to what has been reported from analysis of arterial plaques [15]. Lymphocytes were not significantly affected in the cholesterol treated rats as observed. However, the findings from a study carried out by Van der Wal et al., [31] suggested a predominance of lymphocytes in venous compared with arterial plaques.

A very important step early in atherogenesis is the infiltration of monocytes from the circulation into atherosclerosis-prone vessels [19]. Classical monocytes which represent the largest population of monocytes are important scavenger cells, while non-classical monocytes, often referred to as pro-inflammatory are secondary to their mobilization in disease and secretion of important inflammatory cytokines [12] (i.e., TNF-α). Our findings indicate increased population of monocytes in the groups given low dose of the extract as well as the standard drug atorvastatin. This may be a consequence of the early stage of atherogenesis which is necessary due to the pro-inflammatory nature of the monocytes. The new tripartite view of monocytes subpopulations has shifted attention from the inflammatory characteristics of non-classical monocytes and supports a more significant role for intermediate monocytes in inflammation. Further, unique functions in angiogenesis, production of reactive oxygen species, and patrolling behavior have been attributed to CD16^+^ monocytes [22], [33]. Existence of foamy monocytes in blood was confirmed by other studies in both mice and humans with hyperlipidemia [11], [26]. Nevertheless, it remains unknown when and how foamy monocytes are formed in the blood of mice fed with western high fat diet. Furthermore, the evidence for direct contributions of foamy monocytes to atherosclerosis particularly nascent atherosclerosis in which recent studies showed that monocytes recruitment played a significant role [28] as we also observed, is still lacking.

Histological analysis of the aorta of the experimental rats show that cholesterol administration caused degeneration of the internal elastic lamina in the group fed with the diet only, vascular ulceration and stenosis in the aorta and heavy perivascular infiltrates of inflammatory cells. This was however not observed in the groups given the extract as well as the standard drug. The integrity of the collagen and elastic fibres where also maintained in the groups treated with the extract and the standard drug. Thus, the administration of M. charantia or atorvastatin was effective in ameliorating the deleterious effect of high cholesterol to a very appreciable degree or a near normal condition. This also suggests a similarity in the activity of the extract and that of the standard drug. The results of our present study is in agreement with other studies which state that dietary cholesterol is a major contributor to the elevation of plasma cholesterol which eventually leads to increase serum and aortic tissue cholesterol and as such predisposes to aortic atherosclerosis [2].

## 5 Conclusion

Results from this study indicate that the lowering of oxidative stress level, mediation of inflammation and improvement in the histological features of the aorta of the experimental rats corroborates the anti-atherosclerotic potential of *M. charantia* extract, which could be compared to that of the standard drug. However, more research still needs to be conducted to determine the safety or otherwise of the extract in the biological system.

## Author statement

Dear Editor,

We the authors express our gratitude for the useful comments and suggestions, which in no small measure has made the manuscript better. We also appreciate the reviewers for their valuable criticisms and comments. We have modified the manuscript in accordance with the useful comments and suggestions. The modifications are highlighted in the manuscript.

Thank you for making the revised manuscript a better version.

Kingsley Omage, Ph.D.

## Declaration of Competing Interest

We wish to confirm that there are no known conflicts of interest associated with this publication and there has been no significant financial support for this work that could have influenced its outcome.
